# Study protocol: a randomised controlled trial comparing the long term effects of isolated hip strengthening, quadriceps-based training and free physical activity for patellofemoral pain syndrome (anterior knee pain)

**DOI:** 10.1186/s12891-015-0493-6

**Published:** 2015-02-25

**Authors:** Alexandra Hott, Sigurd Liavaag, Niels Gunnar Juel, Jens Ivar Brox

**Affiliations:** 1 Department of Physical Medicine and Rehabilitation, Sorlandet Hospital Kristiansand, PO box 416, 4604 Kristiansand, Norway; 2 Department of Orthopedic Surgery, Sorlandet Hospital Arendal, PO box 783 Stoa, 4809 Arendal, Norway; 3 Department of Physical Medicine and Rehabilitation, Oslo University Hospital-Ullevål, PO box 4956, Nydalen 0424 Oslo, Norway

**Keywords:** Patellofemoral pain syndrome, Anterior knee pain, Exercise therapy, Hip strengthening

## Abstract

**Background:**

Patellofemoral pain syndrome (PFPS), also known as Anterior Knee Pain, is a common cause of recurrent or chronic knee pain. The etiology is considered to be multifactorial but is not completely understood. At the current time the leading theory is that pathomechanics in the patellofemoral joint leads to PFPS. Traditionally, conservative treatment has focused on improving strength and timing in the quadriceps muscles. In recent years, evidence has been accumulating to support the importance of hip control and strengthening in PFPS. Two recent studies have shown promising results for hip strengthening as an isolated treatment for PFPS. The aim of this randomised contolled trial (RCT) is to compare isolated hip strengthening to traditional quadriceps-based training and a control group with free physical activity.

**Methods/Design:**

An observer-blinded RCT will be performed. We intend to include 150 patients aged 16–40 years, referred from primary care practitioners to the department of Physical Medicine and Rehabilitation in Kristiansand, Norway for PFPS with more than three months duration. Patients meeting the inclusion criteria will be randomised using opaque sequentially numbered sealed envelopes to one of three groups: isolated hip strengthening, quadriceps based training, or a control group (free physical activity). All groups will receive standardized information about PFPS formulated with the intention to minimize fear avoidance and encourage self-mastery of symptoms. Standardized exercises will be performed under supervision of a study physiotherapist once per week in addition to home training two times per week for a total of six weeks. The primary outcome measure will be the Anterior Knee Pain Score (AKPS) at three and 12 months. Secondary outcome measures will include Visual analogue scale (VAS) for pain, hip abductor and quadriceps strength, the generic EuroQol (EQ-5D), Hopkins Symptom Checklist (HSCL), Knee self-efficacy score and Tampa score for Kinesiophobia.

**Discussion:**

This trial will help to elucidate the role of hip and quadriceps strengthening in the treatment of PFPS. Information as to the role of anxiety and depression, kinesiophobia and self-efficacy will be collected, also as regards prognosis and response to exercise therapy.

**Trial registration:**

ClinicalTrials.gov reference: NCT02114294.

## Background

Patellofemoral pain syndrome (PFPS), also known as Anterior Knee Pain, can be defined as pain behind or around the knee cap (patella), provoked during loading of the knee in flexion or extension, in the absence of other specific pathology of the knee joint [1]. It is one of the most common causes of pain in the lower extremity, with the reported prevalence ranging from 7% to as high as 26%, although the true incidence and prevalence of PFPS have not been adequately studied [2-4]. Despite being commonly regarded as a benign and self-limiting condition, several long-term studies show that as many as 73-96% of patients with PFPS have continued pain longer than four years after diagnosis [5-8].

The etiology of PFPS is not completely understood, and is considered to be multifactorial [1,9]. The primary theory at the current time is that patellofemoral malalignment and maltracking (pathomechanics) result in PFPS [10]. Suggested mechanisms causing PFPS are overload, patellar maltracking/malalignment and imbalances in muscle strength and contraction [1,9,11,12]. Factors such as fear avoidance (kinesiophobia) and catastrophizing may be contributing factors [13-15]. Central neurological mechanisms such as sensitization or neuropathic pain also could be possible mechanisms influencing the pain experienced in some patients [16-18].

Several studies have underlined the importance of the quadriceps muscle function including timing and/or activity of the vastus medialis obliquus (VMO) [19-21] although the evidence is conflicting [22]. Traditionally, conservative treatment principles have therefore focused mainly on training strength, balance and timing in quadriceps muscles, especially VMO [1,23,24], often in combination with other modalities such as stretching, taping, and orthotics [1,23,25]. There is some evidence for a short-term effect of quadriceps-based training [23]. However, long term results after quadriceps-based training may be poorer, with up to 80% still reporting pain at five-year follow-up in one study [6].

In recent years there has become an increased focus on the importance of hip strength and control in PFPS. Several studies indicate that women with PFPS have altered hip kinematics during more demanding loading such as running, jumping and landing [26-28]. Studies using dynamic MRI suggest that increased femoral internal rotation results in increased lateral patellar displacement and resultant increased stress in the patellofemoral joint [29,30]. Women with PFPS have been found to be weaker in hip abduction and external rotation compared to healthy controls, although a recent systematic review raises the question as to the causality of the relationship [31,32].

More recent studies have reported promising results when strengthening hip abduction and external rotation on women with PFPS are used prior to [33], or in addition to [34-36], quadriceps-based training, and a recent systematic review concludes that proximal exercises are effective in treating PFPS [37]. A newly published large RCT compared quadriceps-based training to hip and core strengthening and found no significant differences in outcome, but an earlier effect of proximal exercises [38]. Posterolateral hip strengthening has only recently been studied as an isolated treatment for PFPS, and results are promising [39,40]. Much is still unclear regarding the role of proximal muscular strength and control in PFPS. Most published studies are relatively small and have methodological limitations. A newly published systematic review concludes with the need for more research to further evaluate the possible effects of different exercise therapy modalities on PFPS [41].

### Aims

The aim of this study is to compare the effect of isolated hip strengthening exercises, traditional quadriceps based exercises and free physical activity in patients with patellofemoral pain syndrome. The primary outcome is the Anterior Knee Pain Score (AKPS) at three months and one year. Our null-hypothesis is that there is no difference between the three groups for primary or secondary outcomes, measured at three months and one year.

## Methods/Design

### Trial design

**Figure 1:**
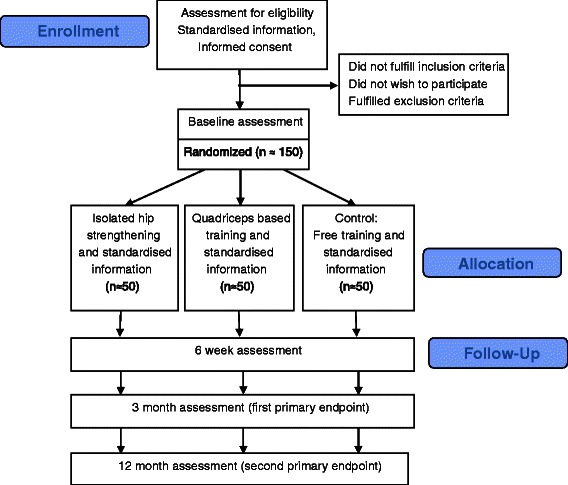
**Patient flow through the study.**

This is an observer-blinded randomised controlled trial (RCT) with first primary endpoint at three months, and second primary endpoint at one year (Figure 1: diagram).

### Ethics

Ethics approval for this study has been received from the Ethics Committee Health Region Southeast, Norway.

### Participants

Participants will be recruited from general practitioners, chiropractors, manual therapists and from departments of orthopedic surgery, rheumatology and physical medicine and rehabilitation. To increase awareness of the trial, potential sources of referral will receive regular written information on the ongoing trial, and general practitioners will be invited to attend lectures on knee complaints with a focus on the current study.

All potential participants will be screened to determine their eligibility according to the following inclusion and exclusion criteria. For inclusion, patients should be 16–40 years of age and have at least three months history of peri- or retropatellar pain with worst pain intensity during previous week of VAS 3 or more. The pain should be provoked by at least two of the following activities: Stair ascent or descent, hopping, running, prolonged sitting, squatting or kneeling. On clinical exam, pain should be present during one of the following: Compression of the patella, palpation of the patellar facets. In patients with bilateral pain the worst knee will be included, and presence of bilateral pain will be documented. One specialist in Physical Medicine and Rehabilitation (PM&R) will perform clinical examinations of all patients. Possible candidates will have a plain x-ray and MRI of the knee joint performed, if this has not already been performed within the previous six months.

Exclusion criteria include clinical, x-ray and MRI findings indicative of meniscal or other intra-articular injury, injury to or increased laxity of cruciate or collateral ligaments, or other pathology including: osteoarthritis, Osgood-Schlatter or Sinding-Larsen-Johanssen syndrome, jumpers knee, or of significant knee joint effusion, significant pain from hip or lumbar spine on clinical evaluation, with potential for causing referred pain to the knee, or hindering the patient’s ability to perform the prescribed exercises, recurrent patellar subluxation or dislocation, previous surgery to the knee joint, NSAID or cortisone use over an extended period of time, having suffered trauma to the knee joint judged during clinical evaluation to have a significant effect on the presenting clinical condition. Patients having received physiotherapy or other similar treatment for patellofemoral pain syndrome within the previous three months will also be excluded.

### Randomisation

Patients who fulfill the inclusion criteria and consent to take part in the trial after they have received the standardized oral and written information, will be randomized to receive hip strengthening (H), quadriceps-based training (Q), or control group (C). Sealed opaque randomization envelopes with a study-specific patient number will be supplied by an external statistician. The randomization sequence is computer-generated with randomization blocks of a variable size which is unknown to any of the research team. A nurse not otherwise involved in the research study will take the sealed opaque numbered envelopes in order, by number, and deliver the correct envelope to the treating physiotherapist. The envelope contains a piece of paper which is labeled with the same patient specific number, plus the group assignment (H, Q or C). The group assignments will be communicated for the purpose of data analysis in coded form. The code will only be revealed when the data analysis is complete.

### Interventions

#### Standardized information

All groups receive standardized oral and written information from the Physical Medicine and Rehabilitation (PM&R) specialist at the time of inclusion to the study focusing on understanding of the etiology of PFPS, and especially reassurance as to the benign nature of the condition. The information is formulated with the intention to minimize fear avoidance and encourage self-mastery of symptoms. Advice will be given to stay physically active without excessively provoking knee pain, and types of potential activities will be explored with the patient. Participants will be asked to refrain from seeking other forms of treatment (eg. physiotherapy, shoe inserts, injections, laser, etc.) during the study. The same written and oral information will be repeated by the study physiotherapists at randomization. Standardization of information will be achieved by regular meetings for the research staff before initiation of the study and throughout the study period.

#### Hip strengthening protocol

The hip strengthening exercises consist of side-lying hip abduction, hip external rotation (clam-shell) and prone hip extension. The exercise positions are based on previous studies of hip strengthening [34,39,40], and are chosen to maximally isolate the hip abductors and external rotators, respectively. Initial dosage of 10 × 3 will be used, with a progression up to a maximum 20 × 3, as previous studies on isolate hip strengthening have used a high-repetition approach [39,40], which is also supported by studies examining dose–response relationship in PFPS exercises [42]. Additional resistance will thereafter be provided by weights or elastic tubing if necessary. One session per week will be performed under supervision of the physiotherapist, with two additional sessions performed as home sessions, for a total of three sessions per week. To avoid focus on pain and pain behaviours, dosage will be adjusted on an individualized basis once weekly by the physiotherapist according to level of function, based on principles for operant conditioning [43]. This entails setting quotas of exercise below the patients’ limit of tolerance as opposed to training up to the pain threshold. A dosage will be chosen in which the last repetitions are difficult while still allowing the patient to maintain high quality of movement control throughout the entire program.

#### Quadriceps-based training

The quadriceps exercise regime is based on previous studies [40,44], and chosen to maximally isolate the quadriceps muscles while still approximating a traditional quadriceps strengthening regime. The exercises will consist of straight leg raises in the supine position, prone terminal knee extensions (from 10° flexion to full extension) and a mini-squat (45° flexion) with the back supported against the wall (to reduce stabilizing requirements from the hip muscles). The timing, duration and progression of the exercise protocol will be matched to the hip strengthening group using the same principles for individualized dosage of exercises described over, adjusted once weekly by the physiotherapist.

#### Control group (free physical activity)

The control group will receive the same standardized information and advice from the specialist as the exercise groups at study inclusion. At randomization, the control group will meet with the study physiotherapist and will again receive standardized information about PFSS and advice about choice and dosage of activities. They will receive no prescribed exercise regime but are encouraged to be physically active according to their own wishes. The purpose of the control group is to assess whether specific hip strengthening or quadriceps-based training is better than free physical activity. This is important if there is no difference in effectiveness between hip strengthening and quadriceps-based training and also for assessing whether any of the specific training regimens are better than free physical activity.

### Outcome assessment

Baseline data will include gender, age, height, weight, body-mass index, level of physical activity, unilateral vs. bilateral symptoms, duration of symptoms, work status, whether on sick leave or disability, highest level of education achieved, use of pain or anti-inflammatory medication, a plain film x-ray including skyline view, and MRI examination of the knee joint (previous examination within 6 months of baseline will be accepted, assuming no significant change in symptoms has occurred during this period). We will also record the Beighton score with regard to joint mobility [45].

Blinded observers will assess all participants at baseline, three months and one year after inclusion. Pain and function, health related quality of life, activity level, strength, self-efficacy, anxiety and depression, number of pain regions, and kinesiophobia will be assessed by standardised, validated questionnaires at each time point. At six weeks, a mid-term assessment will be performed including pain and function (AKPS and VAS) and strength measurements. The treating physiotherapists will be blinded to all baseline data. Due to the nature of the study, blinding of the patients and physiotherapists to the intervention is not possible, but patients’ expectations about the effectiveness of each intervention will be assessed at baseline.

#### Primary outcome measure

The main hypothesis in the present study will be examined at three and 12 months using the Anterior Knee Pain Scale (AKPS) as the primary outcome measure. This is a self-report questionnaire consisting of 13 questions to assess severity of pain and disability [46]. It has been validated for use in this patient population [47]. The categories within each question are weighted, and the responses are summed for an overall index where 100 represents perfect function and 0 represents worst possible function. The minimal clinically important difference (MCID) was reported to be 10 points in a previous study [47]. This questionnaire will be translated to Norwegian and validated according to standard scientific procedures with approval from Kujala et al. [46].

#### Secondary outcome measures

Include a range of standardised, generic self-report measures including VAS for pain, global score of change, step-down test, strength and endurance testing, quality of life, kinesiophobia, anxiety and depression, self-efficacy, number of pain regions, and activity level.

A 10-cm Visual Analog Scale (VAS) will be used to measure pain, where 0 corresponds to no pain and 10 corresponds to worst imaginable pain. The scale is validated for use with patellofemoral pain [47]. We will record VAS for assessment of usual pain the past week (VAS-U) and worst pain during the past week (VAS-W). For these measurements a MCID of 2 has been reported [47].

An 18-point Likert scale for measuring patients’ global assessment of change compared with baseline will be carried out at three and 12 months. The scale ranges from −9 (maximum deterioration) to +9 (maximum improvement) [48].

According to Loudon et al. [49] the step-down (from a 20 cm step) is the only functional test which discriminates between PFSS patients and normal controls. The step down as performed according to standardised instruction will be used to quantify changes in patients’ function. The measure is number of repetitions in 30 seconds.

Isometric strength will be measured for hip abduction, hip external rotation and knee extension. Strength will be measured with a force gauge (MuscleLab Force Gauge 300 kg) using stabilizing straps to ensure correct positioning of the patient, according to the current standard for studies of this type. Standardized positions and procedures are based on techniques proposed by Caldwell et al. [50] and Thorborg et al. [51]. Research personnel will be trained in the procedures by qualified researchers at University of Agder, Norway.

A novel test for hip abduction endurance has been developed for use in this study. A parallel article will be published quantifying normal values for this test, values for PFPS patients (men and women), its inter- and intra-rater reliability, validity compared to strength testing with force measurer, and its predictive value for clinical response to hip strengthening in PFPS patients. Testing is carried out in side-lying position with a 5 kg weight around the ankle of the upper leg, which is in 0-10° hip extension and abducted to 30° above the horizontal plane. Time successfully held in target area is measured in seconds. All participants in the study will be tested at baseline, six weeks, three and 12 months. Parallel with the RCT, normative data will be collected in a group of 75 healthy controls of both sexes from 16–40 years old.

Euro-Qol – 5 Dimensions (EQ-5D) is a questionnaire used to measure health related problems and quality of life [52]. The questionnaire is widely used both internationally and in Norwegian [53]. EQ-5D-5 L includes five questions about mobility, usual activities, self care, pain and discomfort, and anxiety and depression, each has five possible answers. The results are translated to a single summary index value through the use of a table [54]. In addition the subject scores his/her overall health on a 0–100 scale (EQ-VAS).

The Tampa scale for kinesophobia (TSK) is a 13 –item questionnaire aimed at the assessment of fear of movement/re-injury. Each item is scored on a 4-point Likert scale with alternatives ranging from strongly disagree (0) to strongly agree (4). This gives a possible total score range from 0 to 52. The TSK is translated to Norwegian and a cross-cultural adaptation and validation study showed satisfactory validity and reliability in a Norwegian population with sciatica [55].

The Hopkins Symptom Checklist (HSCL) is a symptom inventory which measures symptoms of anxiety, depression and somatisation, originally consisting of 58 items [56]. Comparisons of different versions of the HSCL indicate that a shorter version (HSCL-10) perform almost as well as the full version, also in the Norwegian population [57]. The scale for each question includes 4 categories of response ranging from 1 (not at all) to 4 (extremely). The average HSCL score is calculated by dividing the total score by ten (number of items). In accordance with recommendations by the Regional Ethical Committee, patients who score >2.00 on HSCL-10 (with suspected clinical depression or anxiety disorder that warrants treatment) will receive an offer of referral to psychologist or psychiatrist for follow-up.

Self-efficacy will be measured using the Knee Self-Efficacy Score (K-SES). This score is originally developed in both Swedish and English for measuring self-efficacy in patients with anterior cruciate injury [58] and will be translated to Norwegian for the purpose of this study. K-SES is a self-administered instrument consisting of in total four sections in which patients score how certain they are about specific activities currently and in the future. The K-SES has not been validated for use in PFSS patients, but we consider that it is preferable to a more general self-efficacy score.

An adaptation of the Standardized Nordic Questionnaire [59] will be used to register the number of painful areas. This modified questionnaire is used in a recent Norwegian study [60] which showed a high prevalence of patients with more than one pain site in a Norwegian population, and that there was a strong correlation between increasing number of pain sites and decreasing functional ability.

Activity level will be monitored using a multi-sensor which includes an accelerometer (SenseWear Pro_2_ Armband) using the techniques validated in a study by Berntsen et al. [61]. Study participants will wear the monitor continuously for one week at baseline, three months and 12 months follow-up.

Compliance will be registered weekly by the treating physiotherapist, recording number of completed home sessions and sessions with the physiotherapist. We will also register use of pain medication including non-steroidal anti-inflammatory drugs, sick leave from work, and whether the patient has sought other treatment (type and number).

### Sample size

The power and sample size calculations are based on a one-way Analysis of Variance (ANOVA) model at follow-up that does not take into account adjustment for the baseline values. Based on previous studies, standard deviation of AKPS and VAS is assumed to be 13.5 and 2.25, respectively. MCID for AKPS or VAS is set at 10 and 2, respectively [47]. With these assumptions 27 patients are required in each of the treatment groups (in total 81 patients for all three groups) to obtain 80% statistical power with 5% significance level for AKPS, and 19 in each group for VAS. To take into account possible missing data or drop-outs, a minimum of 35 patients will be included in each group. We will therefore include a minimum of 105 patients in total in this study but intend to include as many as 50 in each group (150 in total) to improve the power of the study considering the assumed heterogeneity of the study population.

### Planned statistical analysis

All eligible patients, regardless of their compliance with the protocol (analysis by intention-to-treat) will be included in the main analysis.

To assess the primary endpoints of the study after three months and one year the differences between the three treatment groups will be analysed with an Analysis of Covariance model using the baseline value as one of the covariates. Assumptions of the model will be checked. To analyse the time course repeated measures will be analysed using linear mixed models. The influence of factors including compliance, muscle strength, psychological factors, joint hypermobility and baseline pain levels will also be examined with regard to treatment response.

A blinded statistical analysis and review of the outcome data is planned [62]. Only after the writing committee members have agreed that there will be no further changes in the interpretation, the randomisation code will be broken, and the manuscript of the final study will be published.

## Discussion

PFPS is a common cause of knee pain in which there are significant levels of recurring or chronic problems. The etiology is not completely understood, and heterogeneity and existence of subgroups has been proposed by many as an explanation for varying results of treatment [1,63]. Traditionally the focus has been on quadriceps strengthening, often as a part of a multimodal approach [23]. Two recent studies have shown promising results for isolated hip strengthening as a method of treating PFPS [39,40]. These studies are relatively small and have methodological weaknesses. A newly published multi-center RCT found no significant difference in outcomes between hip and core strengthening compared with knee exercises [38]. The current study aiming for a sample size of 150 patients will be one of the largest RCTs in this field. The inclusion of a control group receiving standardized information and free physical activity is important. Two recent studies show a superior effect of specific exercises compared to patient education [64,65], but there is a need for more knowledge regarding the effectiveness of specific training compared to patient education and patient-directed free physical activity. In addition, other possible contributing factures, such as anxiety and depression, kinesiophobia, self-efficacy and health-related life quality, will be assessed. Existing data on the relevance of these factors in PFPS is limited but may be of importance with respect to response to specific exercise therapy.

## Abbreviations

PFPS: Patellofemoral pain syndrome
VMO: Vastus medialis obliquus
PM&R: Physical medicine and Rehabilitation
RCT: Randomised controlled trial
AKPS: Anterior knee pain score
VAS: Visual analog scale
MCID: Minimal important clinical difference
EQ-5D: Euro-Qol 5D
TSK: Tampa scale for kinesiophobia
HSCL: Hopkins symptom checklist
K-SES: Knee self-efficacy score
